# Timing for Radiotherapy Initiation After Dental Extraction and Risk of Osteoradionecrosis in Head and Neck Cancer Patients: A Systematic Review and Meta‐Analysis

**DOI:** 10.1002/wjo2.70097

**Published:** 2026-03-15

**Authors:** Nuo‐Zhou Liu, Yi‐Xuan Liu, Zhe Li, Jun Ma, Na Liu

**Affiliations:** ^1^ Department of Radiation Oncology Sun Yat‐sen University Cancer Center, State Key Laboratory of Oncology in South China, Collaborative Innovation Center for Cancer Medicine, Guangdong Key Laboratory of Nasopharyngeal Carcinoma Diagnosis and Therapy Guangzhou People's Republic of China; ^2^ Department of Prosthodontics Guanghua School of Stomatology, Hospital of Stomatology, Sun Yat‐sen University, Guangdong Provincial Key Laboratory of Stomatology Guangzhou China; ^3^ Department of Experimental Research State Key Laboratory of Oncology in South China, Guangdong Key Laboratory of Nasopharyngeal Carcinoma Diagnosis and Therapy, Sun Yat‐sen University Cancer Center Guangzhou China

**Keywords:** dental extraction, head and neck cancer, osteoradionecrosis, radiation, radiotherapy

## Abstract

**Background:**

Head and neck cancer (HNC) patients are advised to undergo dental extraction of teeth with poor prognosis prior to radiotherapy (RT) initiation to prevent osteoradionecrosis (ORN). However, insufficient healing time may result in persistent extraction wounds during RT, which constitute a potential risk factor for ORN, whereas excessive delay in RT initiation may compromise cancer control.

**Objectives:**

To identify an optimal healing period between pre‐RT dental extraction and RT initiation to minimize ORN risk in HNC patients.

**Methods:**

This systematic review and meta‐analysis were conducted in accordance with the Preferred Reporting Items for Systematic Reviews and Meta‐Analyses (PRISMA) guidelines and the Meta‐analysis of Observational Studies in Epidemiology (MOOSE) framework, with a pre‐specified PROSPERO registration (ID: CRD42024586754). The Newcastle–Ottawa Scale was employed to assess the quality of included studies. Patients were stratified into ≤ 7 days versus > 7 days and ≤ 14 days versus > 14 days groups based on the interval between dental extraction and RT initiation. Subgroup analyses were performed by geographic population.

**Results:**

Eight retrospective cohort studies involving 47,527 HNC patients and 2863 ORN cases were included. No significant association was observed between the timing of pre‐RT dental extraction and ORN risk in the overall population (OR = 0.99, 95%CI: 0.67–1.47), with significant heterogeneity (*I*² = 92%, *τ*² = 0.2079, *p* < 0.01). In Western populations, patients initiating RT within < 7 days or < 14 days after dental extraction demonstrated significantly elevated ORN risk (OR = 3.07, 95%CI: 1.52–6.21 and OR = 2.01, 95%CI:1.10–3.68, respectively).

**Conclusion:**

An insufficient healing interval between dental extraction and RT initiation may significantly increase ORN risk, particularly in specific population subgroups.

## 1 Introduction

Head and neck cancer (HNC) is the sixth most diagnosed malignancy globally, accounting for over 890,000 new cases and 81,000 deaths annually, imposing substantial socioeconomic burdens [1, 2].

Advances in intensity‐modulated radiation therapy (IMRT), combined with systemic therapies and surgical techniques, have significantly improved long‐term survival rates [3]. Consequently, comprehensive management of treatment‐related complications—including dental care, nutritional support, mental health interventions, and swallowing rehabilitation—has gained increasing importance in improving quality of life (QoL), treatment compliance, and clinical outcomes [4, 5, 6, 7].

Dental comorbidities in HNC patients arise not only from pre‐existing poor oral hygiene and tumor‐induced structural/functional damage, but also from radiation toxicities such as mucositis, xerostomia, trismus, and caries [8, 9]. Current guidelines strongly advocate multidisciplinary dental evaluation before, during, and after RT to mitigate complications [4, 8, 10, 11]. Among these, osteoradionecrosis (ORN)—defined as non‐healing irradiated bone without tumor recurrence—represents a severe late complication that compromises QoL and may necessitate RT discontinuation [12, 13, 14, 15].

Given the absence of standardized ORN treatments, preventive strategies through risk factor identification are critical [12, 16]. Post‐RT dental extractions are widely recognized as a modifiable ORN risk factor [17, 18], prompting recommendations for pre‐RT removal of non‐restorable teeth [8]. However, the optimal timing of pre‐RT extractions remains controversial. If the RT starts too early when the wound is not properly healed, the defects in oral mucosa can persist for a long period of time and become a potential risk for ORN [19]. Routinely, the starting date of RT is often 2–3 weeks after dental extrication to allow wound healing, which is supported by multiple publications including US National Comprehensive Cancer Network (NCCN) Guidelines [3, 8]. However, the duration (2–3 weeks or even more) of RT delay after dental extraction seems primarily based on clinical experience and opinions with not enough supporting evidence.

Moreover, previous studies have also indicated that several HNC types can progress very fast, where a 6‐week delay in RT after surgery can harm patients' overall survival [20, 21]. A trade‐off should be considered between ORN risk and cancer control for those HNC patients needing pre‐RT dental extraction.

Hence, the preliminary aim of this study was to review the existing evidence and identify a proper healing period for RT onset after dental extraction for HNC patients to avoid ORN occurrence premising timely initiation of RT and cancer control.

## 2 Methods

This systematic review and meta‐analysis was conducted in accordance with the Preferred Reporting Items for Systematic Reviews and Meta‐Analyses (PRISMA) guidelines and the Meta‐analysis of Observational Studies in Epidemiology (MOOSE) framework, with a pre‐specified PROSPERO registration (ID: CRD42024586754). Institutional review board approval and patient informed consent were not required as this study analyzed aggregated data from previously published studies.

### 2.1 Information Sources and Search Strategy

A systematic search of PubMed/MEDLINE, Embase, Web of Science, ClinicalTrials.gov, and the Cochrane Library was conducted from database inception to September 1, 2024, with no geographic restrictions. The detailed search strategies are provided in Supporting Information: S1. Additionally, we performed manual screening of reference lists from relevant articles to supplement the electronic search results.

### 2.2 Eligibility Criteria

The inclusion criteria were listed as follows: All interventional (randomized clinical trials) and observational studies (cross‐sectional, case–control, or cohort designs) investigating HNC patients undergoing dental extraction before, during, or after radiotherapy (RT) with documented osteoradionecrosis (ORN) incidence. No restrictions on RT modalities (including external beam radiation therapy (EBRT), intensity‐modulated radiation therapy (IMRT), two‐dimensional, three‐dimensional, conformal RT, or brachytherapy). No limitations on HNC tumor subsites or dental extraction locations. Then, we excluded studies lacking direct comparisons of dental extraction timing relative to RT (e.g., studies comparing only pre‐RT vs. post‐RT extractions without temporal stratification). To ensure the quality and stability of statistics, cohorts less than 25 participants in each arm, non‐comparative studies, publications lacking full text availability, or non‐English publications were excluded. Two reviewers (NZ L; YX L) independently checked the search results, and evaluated study eligibility by screening titles and abstracts. All potentially eligible studies underwent full‐text review. Discrepancies were resolved through team consensus after structured discussion.

### 2.3 Data Extraction, Main Outcomes, and Risk of Bias Assessment

Two authors (NZ L; YX L) independently extracted the correlated information on first author, study registration number (if accessible), publication year, study origin and design, cancer type and site, RT parameters (modality, location, dose, and duration), other systemic therapy or surgery, incidence/cases of ORN among different dental extraction time before RT, dental extraction site and total number of extractions. If the actual case number of ORN was not reported, the fully adjusted odds ratio with 95% confidence interval (OR, 95%CI) was utilized.

Newcastle–Ottawa Scale (NOS) score with 8 distinct domains, including representativeness of the exposed cohort, selection of the nonexposed cohort, ascertainment of exposure, outcome of the interest not present at the start of the study, comparability of the cohort, assessment of outcome, follow‐up duration, and adequacy of follow‐up of the cohorts, was used to assess the risk of bias in primary observational studies. Cochrane Risk of Bias (ROB) grading system for interventional studies with five domains, including randomization, intended interventions, missing outcome data, outcome measurement, and selection of report was used to assess primary interventional study. Two reviewers (NZ L; YX L) independently conducted all assessments. Discrepancies were resolved through team consensus after structured discussion. Detailed bias assessment results are presented in the Results section.

### 2.4 Statistical Analysis

The OR was selected as the effect measure to mitigate potential bias from small sample sizes, given the reported 5%–7% incidence of ORN in RT‐treated populations. To optimize statistical power and clinical applicability [11, 22], we stratified the interval between last dental extraction and RT initiation into: ≤ 7 days versus > 7 days and ≤ 14 days versus > 14 days. Pooled OR and 95% CI for binary outcomes (ORN, a dichotomous outcome) between different time of dental extraction before RT were calculated by random or fixed‐effects models. *Q* tests and *I* ^2^ statistic were used to assess the statistical heterogeneity between each primary study, where common/fixed‐effects models with Mantel–Haenszel methods were performed when there was no significant heterogeneity (*Q* test *p* > 0.05; *I* ^2^ < 50%), otherwise, random‐effects models with DerSimonian–Laird method were applied. Subgroup analyses stratified by geographic origin (Asian vs. Western populations) were performed. Sensitivity analyses involved iteratively excluding individual studies to evaluate result robustness. We assessed publication bias by funnel plot (visually) and the Egger test to test the symmetry of funnel plot (statistically). All analyses were conducted in R v4.0.5 (www.R-project.org) with RStudio v1.4.1, using two‐tailed *p* < 0.05 as the significance threshold.

## 3 Results

### 3.1 The Selection Procedure, Characteristics and Risk of Bias of Included Studies

**Figure 1:**
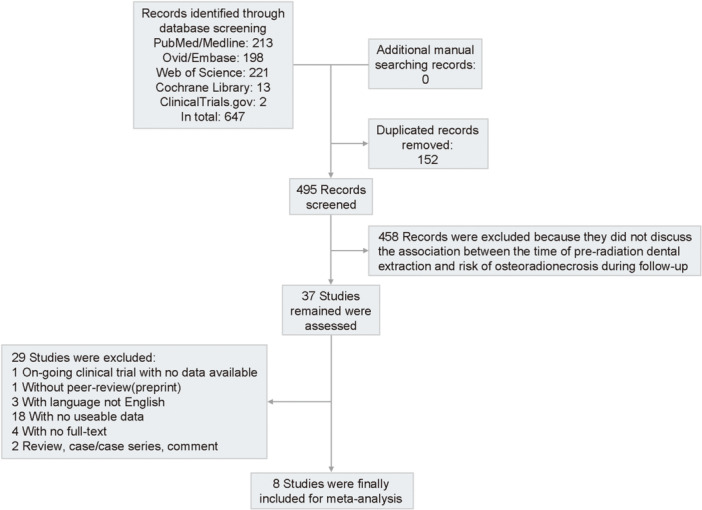
Flow diagram for study selection.

A total of 647 studies were initially identified from PubMed/MEDLINE, Ovid/Embase, Web of Science, Cochrane Library, and ClinicalTrials.gov through title/abstract screening (Figure 1). After excluding duplicates and studies with unavailable or nonsynthesizable data, eight retrospective cohort studies were included for full‐text analysis [19, 23, 24, 25, 26, 27, 28, 29]. The baseline characteristics of the included studies are summarized in Supporting Information: Table 1. These studies enrolled 47,527 participants (2863 ORN cases) aged ≥ 18 years, all diagnosed with HNC and having undergone ≥ 1 dental extraction prior to RT. Risk of bias was assessed using the NOS criteria (Supporting Information: Table 2), with NOS scores ranging from 6 to 9 (indicating moderate‐to‐high methodological quality).

### 3.2 Timing for Pre‐RT Dental Extraction and Risk of ORN

**Figure 2:**
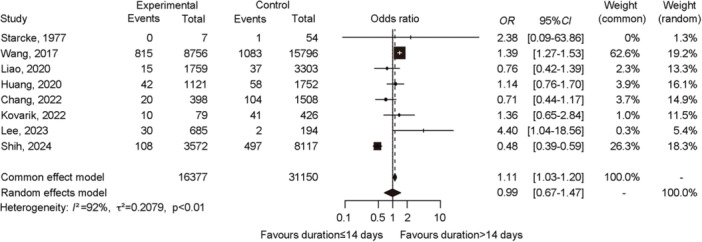
Forest plot for the association between timing for RT initiation (≤ 14 days vs. > 14 days) and risk of osteoradionecrosis. CI, confidence interval; OR, odds ratio; RT, radiotherapy.

**Figure 3:**
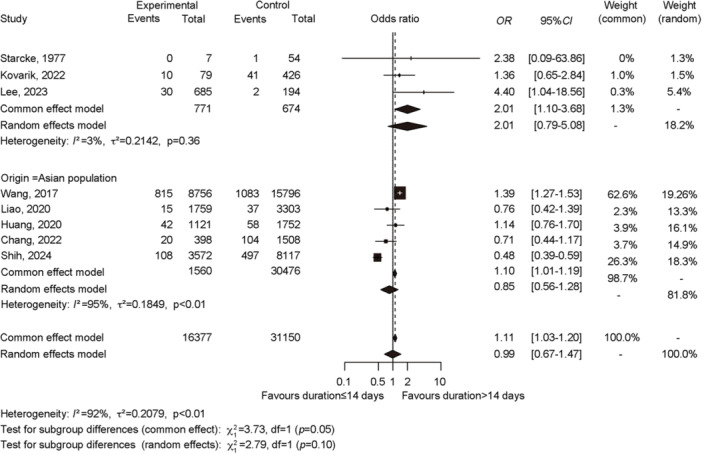
Forest plot for the association between timing for RT initiation (≤ 14 days vs. > 14 days) and risk of osteoradionecrosis stratified by study origin. CI, confidence interval; OR, odds ratio; RT, radiotherapy.

**Figure 4:**
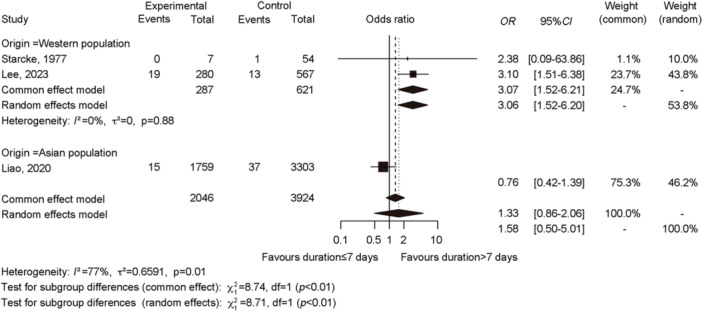
Forest plot for the association between timing for RT initiation (≤ 7 days vs. > 7 days) and risk of osteoradionecrosis stratified by study origin. CI, confidence interval; OR, odds ratio; RT, radiotherapy.

With significant heterogeneity (*I* ^2^ = 92%, *τ* ^2^ = 0.2079, *Q* test *p* < 0.01), random‐effects model indicated that the timing for pre‐RT dental extraction was not associated with ORN risk (OR = 0.99, 95%CI: 0.67–1.47) while common‐effects model still indicated a higher ORN risk for HNC patients receiving dental extraction less than 14 days before RT initiation (OR = 1.11, 95%CI: 1.03–1.20) (Figure 2). No significant publication bias was found (Egger test *p* = 0.613, Supporting Information: Figure 1). To investigate heterogeneity, leave‐one‐out sensitivity analysis identified the study by Shih et al. (2024) [29] as the primary source (Supporting Information: Figure 2). Given differences in HNC subtype prevalence and RT protocols between Western and Asian populations, we performed subgroup analyses (Figure 3). Western populations initiating RT within < 14 days post‐extraction exhibited significantly elevated ORN risk (OR = 2.01, 95%CI: 1.10–3.68; *I*² = 3%, *τ*² = 0.2142, *Q*‐test *p* = 0.36). To refine temporal thresholds, we stratified the interval into ≤ 7 versus > 7 days (Figure 4). Western populations with ≤ 7‐day intervals had significantly higher ORN risk (OR = 3.07, 95%CI: 1.52–6.21; low heterogeneity), despite limited study numbers.

## 4 Discussion

This systematic review and meta‐analysis demonstrated that healing periods of < 7 days and < 14 days after dental extraction were associated with a significantly higher risk of ORN in Western HNC patients undergoing RT, whereas no such association was observed in the overall cohort or Asian populations. The methodological quality of included studies was deemed acceptable, as assessed by the NOS grading system.

The dental status of HNC patients is frequently suboptimal, due not only to tumor‐related effects but also to treatment sequelae, particularly RT [30, 31, 32, 33]. Common RT‐induced oral complications include mucositis, xerostomia, and taste alterations, with ORN representing one of the most severe irreversible late effects that profoundly impair QoL in HNC survivors [13]. Many attempts have been made to address ORN, like recognition and modification of pre‐existing risk factor, construction of ORN risk predictive model, more frequent dental visit, and Multi‐Disciplinary Treatment (MDT) team with experienced dentists [34, 35, 36, 37]. Given that RT‐induced structural tooth damage, salivary dysfunction, and periodontal deterioration may result in technically complex and risky post‐RT extractions with elevated ORN risk [31, 32, 38, 39], pre‐RT extraction of non‐restorable teeth has been widely adopted as a cornerstone of prophylactic protocols [3, 8, 10].

Nevertheless, several studies have demonstrated that pre‐radiation dental extraction may fail to prevent ORN or even paradoxically increase its risk, challenging the routine use of this practice [17, 28, 40, 41, 42, 43]. After we carefully reviewed related publications, we found several interpretations of these results and thus conducted this systematic review and meta‐analysis to verify the relationship between pre‐RT dental extraction and ORN incidence. First and foremost, the timing for RT initiation after dental extraction is critically important and can confound the association between pre‐RT dental extraction risk of ORN. Our results indicated that an insufficient waiting (healing) period, especially less than 7 or 14 days after dental extraction, could perhaps significantly increase ORN risk. Thus, pooling data without temporal stratification may yield conflicting conclusions [17, 43]. Pre‐RT extractions are typically performed in patients with pre‐existing poor dentition, which itself constitutes an ORN risk factor and potentially complicates causal inference between extraction procedures and ORN development [44].

Current guidelines recommend pre‐RT dental extraction with a sufficient healing period [3, 8, 10, 45]. However, their definitions for healing interval and non‐retainable teeth remain broad and imprecise, lacking a foundation in evidence‐based medicine. Consequently, our findings provide essential evidence‐based support for pre‐RT dental management. Nevertheless, the optimal healing duration remains controversial, as does the definition of adequate wound healing. Lee et al. [19] reported ORN occurrence in two patients initiating RT 12 days post‐extraction—a timeframe traditionally deemed safe. This discrepancy may stem from the disparate healing requirements: While epithelial socket closure occurs within 1–2 weeks, bone tissue regeneration requires 3–6 months [46, 47]. Delaying RT to await complete bone healing is clinically unfeasible given the rapid progression of many HNC subtypes [20, 21]. So, a trade‐off between wound healing for ORN prevention and cancer control must be carefully considered.

While current guidelines and our findings support pre‐RT dental extraction with a defined healing period [3, 8, 10, 45], overly aggressive extraction protocols are still not recommended, as they may result in unnecessary tooth loss, impaired nutritional status and QoL, reduced compliance to treatment strategy, and even unsatisfied cancer control [48, 49]. This underscores the need for strict criteria to define “non‐retainable teeth”—a decision requiring multidisciplinary evaluation [22]. Different criteria for location‐specific extraction were detailedly reported in a Delphi study [50]. For instance, teeth with severe periodontitis should be extracted prior to RT [41]. However, variability in tumor subsites, RT parameters (dose/fractionation), and baseline oral health complicates individualized decisions for location‐specific pre‐RT dental extraction. Emerging tools like machine learning‐based predictive models for high‐risk teeth and radiation dose tolerance thresholds may help clinical practice [51, 52, 53, 54].

This study has several limitations. Eight retrospective cohort studies were included for analysis with no randomized clinical trials (RCTs), affecting the quality of evidence. However, designing RCT for this topic is quite difficult due to ethical restriction, since current guidelines have already recommended a waiting time for RT initiation after dental extraction. Due to a lack of data, we could not discuss the impact of tooth extraction site and the total number of tooth extraction, which might influence ORN development. Similarly, we did not have a direct opportunity to consider the impact of RT modality, dose, and field design on ORN development in this meta‐analysis despite their established impact on ORN risk [55, 56]. To partially address this, we performed subgroup analyses by study origin, as modern RT technique adoption (e.g., Co^60^ vs. IMRT) and HNC subtype prevalence differ between Asian and Western populations. However, small sample sizes (particularly for < 7‐day intervals) and persistent heterogeneity in Asian studies restrict conclusive interpretations.

## 5 Conclusions

Our findings suggested that an insufficient healing period for pre‐RT dental extraction, especially less than 7 or 14 days, could perhaps significantly increase ORN risk at least in several population settings. Further prospective studies investigating the proper time for RT initiation after dental extraction to minimize the ORN risk and starting RT timely for cancer control at the same time are still in need.

## Author Contributions

Conception and design or analysis and interpretation of data: Nuo‐Zhou Liu and Yi‐Xuan Liu. Drafting of the manuscript or revising it for important intellectual content: Jun Ma and Na Liu. Final approval of the version to be published: Nuo‐Zhou Liu, Yi‐Xuan Liu, Jun Ma, and Na Liu.

## Ethics Statement

No separate ethic review and approval was needed in this secondary study.

## Conflicts of Interest

The authors declare no conflicts of interest.

## Supporting information


**Supplementary Figure 1:** Funnel plot to assess potential bias from publication. **Supplementary Figure 2:** Forest plot for sensitivity analysis (leave‐one‐out) for the association between timing for RT initiation (≤ 14days vs > 14 days) and risk of osteoradionecrosis. **Supplementary Table 1:** Characteristics of included studies for meta‐analysis. **Supplementary Table 2:** Newcastle‐Ottawa Scale (NOS) for risk of bias evaluation.

## Data Availability

Data described in the manuscript, code book, and analytic code will be made publicly and freely available without restriction.

## References

[1] H. Sung , J. Ferlay , R. L. Siegel , et al., “Global Cancer Statistics 2020: GLOBOCAN Estimates of Incidence and Mortality Worldwide for 36 Cancers in 185 Countries,” CA: A Cancer Journal for Clinicians 71, no. 3 (2021): 209–249, 10.3322/caac.21660. 33538338

[2] F. Bray , M. Laversanne , H. Sung , et al., “Global Cancer Statistics 2022: GLOBOCAN Estimates of Incidence and Mortality Worldwide for 36 Cancers in 185 Countries,” CA: A Cancer Journal for Clinicians 74, no. 3 (2024): 229–263, 10.3322/caac.21834. 38572751

[3] J. J. Caudell , M. L. Gillison , E. Maghami , et al., “NCCN Guidelines® Insights: Head and Neck Cancers, Version 1.2022,” Journal of the National Comprehensive Cancer Network 20, no. 3 (2022): 224–234, 10.6004/jnccn.2022.0016. 35276673

[4] E. E. Cohen , S. J. LaMonte , N. L. Erb , et al., “American Cancer Society Head and Neck Cancer Survivorship Care Guideline,” CA: A Cancer Journal for Clinicians 66, no. 3 (2016): 203–239, 10.3322/caac.21343. 27002678

[5] D. N. Margalit , T. Salz , R. Venchiarutti , et al., “Interventions for Head and Neck Cancer Survivors: Systematic Review,” Head & Neck 44, no. 11 (2022): 2579–2599, 10.1002/hed.27142. 35848095 PMC9796901

[6] S. Falek , R. Regmi , J. Herault , et al., “Dental Management in Head and Neck Cancers: From Intensity‐Modulated Radiotherapy With Photons to Proton Therapy,” Supportive Care in Cancer 30, no. 10 (2022): 8377–8389, 10.1007/s00520-022-07076-5. 35513755

[7] K. J. Banda , H. Chu , C. C. Kao , et al., “Swallowing Exercises for Head and Neck Cancer Patients: A Systematic Review and Meta‐Analysis of Randomized Control Trials,” International Journal of Nursing Studies 114 (2021): 103827, 10.1016/j.ijnurstu.2020.103827. 33352439

[8] E. Watson , Z. D. Mojdami , A. Oladega , A. Hope , and M. Glogauer , “Clinical Practice Guidelines for Dental Management Prior to Radiation for Head and Neck Cancer,” Oral Oncology 123 (2021): 105604, 10.1016/j.oraloncology.2021.105604. 34775180

[9] J. B. Epstein , J. Thariat , R. J. Bensadoun , et al., “Oral Complications of Cancer and Cancer Therapy: From Cancer Treatment to Survivorship,” CA: A Cancer Journal for Clinicians 62, no. 6 (2012): 400–422, 10.3322/caac.21157. 22972543

[10] Chinese Head and Neck Cancer Working Group, “ Chinese Society of Clinical Oncology (CSCO) Diagnosis and Treatment Guidelines for Head and Neck Cancer 2018 (English Version),” Chinese Journal of Cancer Research 31, no. 1 (2019): 84–98, 10.21147/j.issn.1000-9604.2019.01.05. 30996570 PMC6433585

[11] D. G. Pfister , S. Spencer , D. Adelstein , et al., “Head and Neck Cancers, Version 2.2020, NCCN Clinical Practice Guidelines in Oncology,” Journal of the National Comprehensive Cancer Network 18, no. 7 (2020): 873–898, 10.6004/jnccn.2020.0031. 32634781

[12] K. O'Dell and U. Sinha , “Osteoradionecrosis,” Oral and Maxillofacial Surgery Clinics of North America 23, no. 3 (2011): 455–464, 10.1016/j.coms.2011.04.011. 21798443

[13] A. Chronopoulos , T. Zarra , M. Ehrenfeld , and S. Otto , “Osteoradionecrosis of the Jaws: Definition, Epidemiology, Staging and Clinical and Radiological Findings. A Concise Review,” International Dental Journal 68, no. 1 (2018): 22–30, 10.1111/idj.12318. 28649774 PMC9378891

[14] F. De Felice , V. Tombolini , D. Musio , and A. Polimeni , “Radiation Therapy and Mandibular Osteoradionecrosis: State of the Art,” Current Oncology Reports 22, no. 9 (2020): 89, 10.1007/s11912-020-00954-3. 32642937

[15] F. Yang , R. J. Wong , K. Zakeri , A. Singh , C. L. Estilo , and N. Y. Lee , “Osteoradionecrosis Rates After Head and Neck Radiation Therapy: Beyond the Numbers,” Practical Radiation Oncology 14, no. 4 (2024): e264–e275, 10.1016/j.prro.2024.02.008. 38649030

[16] A. J. Frankart , M. J. Frankart , B. Cervenka , A. L. Tang , D. G. Krishnan , and V. Takiar , “Osteoradionecrosis: Exposing the Evidence Not the Bone,” International Journal of Radiation Oncology*Biology*Physics 109, no. 5 (2021): 1206–1218, 10.1016/j.ijrobp.2020.12.043. 33412258

[17] P. Balermpas , J. E. van Timmeren , D. J. Knierim , M. Guckenberger , and I. F. Ciernik , “Dental Extraction, Intensity‐Modulated Radiotherapy of Head and Neck Cancer, and Osteoradionecrosis: A Systematic Review and Meta‐Analysis,” Strahlentherapie und Onkologie 198, no. 3 (2022): 219–228, 10.1007/s00066-021-01896-w. 35029717 PMC8863691

[18] J. M. White , N. H. Panchal , C. J. Wehler , et al., “Department of Veterans Affairs Consensus: Preradiation Dental Treatment Guidelines for Patients With Head and Neck Cancer,” Head & Neck 41, no. 5 (2019): 1153–1160, 10.1002/hed.25519. 30620438

[19] J. Lee , K. Hueniken , K. Cuddy , et al., “Dental Extractions Before Radiation Therapy and the Risk of Osteoradionecrosis in Patients With Head and Neck Cancer,” JAMA Otolaryngology–Head & Neck Surgery 149, no. 12 (2023): 1130–1139, 10.1001/jamaoto.2023.3429. 37856115 PMC10587826

[20] E. M. Graboyes , E. Garrett‐Mayer , M. A. Ellis , et al., “Effect of Time to Initiation of Postoperative Radiation Therapy on Survival in Surgically Managed Head and Neck Cancer,” Cancer 123, no. 24 (2017): 4841–4850, 10.1002/cncr.30939. 28841234 PMC5759768

[21] A. Coca‐Pelaz , R. P. Takes , K. Hutcheson , et al., “Head and Neck Cancer: A Review of the Impact of Treatment Delay on Outcome,” Advances in Therapy 35, no. 2 (2018): 153–160, 10.1007/s12325-018-0663-7. 29396681

[22] M. Buglione , R. Cavagnini , F. Di Rosario , et al., “Oral Toxicity Management in Head and Neck Cancer Patients Treated With Chemotherapy and Radiation: Dental Pathologies and Osteoradionecrosis (Part 1) Literature Review and Consensus Statement,” Critical Reviews in Oncology/Hematology 97 (2016): 131–142, 10.1016/j.critrevonc.2015.08.010. 26318095

[23] E. N. Starcke and I. L. Shannon , “How Critical Is the Interval Between Extractions and Irradiation in Patients With Head and Neck Malignancy,” Oral Surgery, Oral Medicine, Oral Pathology 43, no. 3 (1977): 333–337, 10.1016/0030-4220(77)90317-6. 265036

[24] T. H. Wang , C. J. Liu , T. F. Chao , T. J. Chen , and Y. W. Hu , “Risk Factors for and the Role of Dental Extractions in Osteoradionecrosis of the Jaws: A National‐Based Cohort Study,” Head & Neck 39, no. 7 (2017): 1313–1321, 10.1002/hed.24761. 28370713

[25] Y. F. Huang , S. P. Liu , C. H. Muo , C. H. Tsai , and C. T. Chang , “The Association Between Dental Therapy Timelines and Osteoradionecrosis: A Nationwide Population‐Based Cohort Study,” Clinical Oral Investigations 24, no. 1 (2020): 455–463, 10.1007/s00784-019-02866-4. 31111283

[26] P. H. Liao , C. H. Chu , P. L. Tang , P. C. Wu , and T. J. Kuo , “Preradiation Tooth Extraction and Jaw Osteoradionecrosis: Nationwide Population‐Based Retrospective Study in Taiwan,” Clinical Otolaryngology 45, no. 6 (2020): 896–903, 10.1111/coa.13624. 32738824

[27] C. T. Chang , S. P. Liu , C. H. Muo , et al., “The Impact of Dental Therapy Timelines and Irradiation Dosages on Osteoradionecrosis in Oral Cancer Patients: A Population‐Based Cohort Study,” Oral Oncology 128 (2022): 105827, 10.1016/j.oraloncology.2022.105827. 35364549

[28] J. P. Kovarik , I. Voborna , S. Barclay , et al., “Osteoradionecrosis After Treatment of Head and Neck Cancer: A Comprehensive Analysis of Risk Factors With a Particular Focus on Role of Dental Extractions,” British Journal of Oral and Maxillofacial Surgery 60, no. 2 (2022): 168–173, 10.1016/j.bjoms.2021.03.009. 34857411

[29] Y. J. Shih , J. Y. Huang , Y. C. Lai , H. M. Lin , and T. J. Kuo , “Tooth Extraction Within 2 Weeks Before Radiotherapy and Osteoradionecrosis: A Nationwide Cohort Study,” Oral Diseases 30, no. 2 (2024): 575–585, 10.1111/odi.14349. 35951468

[30] J. B. Epstein and A. Barasch , “Oral and Dental Health in Head and Neck Cancer Patients,” Cancer Treatment and Research 174 (2018): 43–57, 10.1007/978-3-319-65421-8_4. 29435836

[31] M. T. Brennan , N. S. Treister , T. P. Sollecito , et al., “Dental Disease Before Radiotherapy in Patients With Head and Neck Cancer,” Journal of the American Dental Association 148, no. 12 (2017): 868–877, 10.1016/j.adaj.2017.09.011. 29173331 PMC5777182

[32] S. B. Jensen , A. M. Pedersen , A. Vissink , et al., “A Systematic Review of Salivary Gland Hypofunction and Xerostomia Induced by Cancer Therapies: Prevalence, Severity and Impact on Quality of Life,” Supportive Care in Cancer 18, no. 8 (2010): 1039–1060, 10.1007/s00520-010-0827-8. 20237805

[33] S. Høxbroe Michaelsen , C. Grønhøj , J. Høxbroe Michaelsen , J. Friborg , and C. von Buchwald , “Quality of Life in Survivors of Oropharyngeal Cancer: A Systematic Review and Meta‐Analysis of 1366 Patients,” European Journal of Cancer 78 (2017): 91–102, 10.1016/j.ejca.2017.03.006. 28431302

[34] K. T. Fitzgerald , C. Lyons , A. England , et al., “Risk Factors Associated With the Development of Osteoradionecrosis (ORN) in Head and Neck Cancer Patients in Ireland: A 10‐year Retrospective Review,” Radiotherapy and Oncology 196 (2024): 110286, 10.1016/j.radonc.2024.110286. 38641259

[35] E. E. Watson , K. Hueniken , J. Lee , et al., “Development and Standardization of An Osteoradionecrosis Classification System in Head and Neck Cancer: Implementation of a Risk‐Based Model,” Journal of Clinical Oncology 42, no. 16 (2024): 1922–1933, 10.1200/JCO.23.01951. 38691822 PMC11500043

[36] S. L. Kelly , J. E. Jackson , B. E. Hickey , F. G. Szallasi , and C. A. Bond , “Multidisciplinary Clinic Care Improves Adherence to Best Practice in Head and Neck Cancer,” American Journal of Otolaryngology 34, no. 1 (2013): 57–60, 10.1016/j.amjoto.2012.08.010. 23218113

[37] N. Beech , S. Robinson , S. Porceddu , and M. Batstone , “Dental Management of Patients Irradiated for Head and Neck Cancer,” Australian Dental Journal 59, no. 1 (2014): 20–28, 10.1111/adj.12134. 24495127

[38] D. Koga , J. Salvajoli , and F. Alves , “Dental Extractions and Radiotherapy in Head and Neck Oncology: Review of the Literature,” Oral Diseases 14, no. 1 (2008): 40–44, 10.1111/j.1601-0825.2006.01351.x. 18173447

[39] I. Saito , T. Hasegawa , Y. Kawashita , et al., “Association Between Dental Extraction After Radiotherapy and Osteoradionecrosis: A Multi‐Centre Retrospective Study,” Oral Diseases 28, no. 4 (2022): 1181–1187, 10.1111/odi.13826. 33650256

[40] N. M. Beech , S. Porceddu , and M. D. Batstone , “Radiotherapy‐Associated Dental Extractions and Osteoradionecrosis,” Head & Neck 39, no. 1 (2017): 128–132, 10.1002/hed.24553. 27473832

[41] D. T. Chang , P. R. Sandow , C. G. Morris , et al., “Do Pre‐Irradiation Dental Extractions Reduce the Risk of Osteoradionecrosis of the Mandible,” Head & Neck 29, no. 6 (2007): 528–536, 10.1002/hed.20538. 17230555

[42] H. Jawad , N. A. Hodson , and P. J. Nixon , “A Review of Dental Treatment of Head and Neck Cancer Patients, Before, During and After Radiotherapy: Part 1,” British Dental Journal 218, no. 2 (2015): 65–68, 10.1038/sj.bdj.2015.28. 25613260

[43] D. H. Moon , S. H. Moon , K. Wang , et al., “Incidence of, and Risk Factors for, Mandibular Osteoradionecrosis in Patients With Oral Cavity and Oropharynx Cancers,” Oral Oncology 72 (2017): 98–103, 10.1016/j.oraloncology.2017.07.014. 28797468

[44] A. Naseer , F. Goode , and T. Doyle , “Osteoradionecrosis ‐ An Old Problem With New Consequences,” Current Opinion in Supportive & Palliative Care 18, no. 1 (2024): 39–46, 10.1097/SPC.0000000000000690. 38170197

[45] H. H. Bruins , D. E. Jolly , and R. Koole , “Preradiation Dental Extraction Decisions in Patients With Head and Neck Cancer,” Oral Surgery, Oral Medicine, Oral Pathology, Oral Radiology, and Endodontology 88, no. 4 (1999): 406–412, 10.1016/s1079-2104(99)70053-3. 10519746

[46] G. Cardaropoli , M. Araújo , and J. Lindhe , “Dynamics of Bone Tissue Formation in Tooth Extraction Sites. An Experimental Study in Dogs,” Journal of Clinical Periodontology 30, no. 9 (2003): 809–818, 10.1034/j.1600-051x.2003.00366.x. 12956657

[47] J. O. Agbaje , R. Jacobs , K. Michiels , M. Abu‐Ta'a , and D. van Steenberghe , “Bone Healing After Dental Extractions in Irradiated Patients: A Pilot Study on a Novel Technique for Volume Assessment of Healing Tooth Sockets,” Clinical Oral Investigations 13, no. 3 (2009): 257–261, 10.1007/s00784-008-0231-7. 18985394

[48] D. J. M. Buurman , C. M. Speksnijder , M. E. Granzier , V. C. M. L. Timmer , F. J. P. Hoebers , and P. Kessler , “The Extent of Unnecessary Tooth Loss Due to Extractions Prior to Radiotherapy Based on Radiation Field and Dose in Patients With Head and Neck Cancer,” Radiotherapy and Oncology 187 (2023): 109847, 10.1016/j.radonc.2023.109847. 37543058

[49] D. J. M. Buurman , A. C. H. Willemsen , C. M. Speksnijder , et al., “Tooth Extractions Prior to Chemoradiation or Bioradiation Are Associated With Weight Loss During Treatment for Locally Advanced Oropharyngeal Cancer,” Supportive Care in Cancer 30, no. 6 (2022): 5329–5338, 10.1007/s00520-022-06942-6. 35278135 PMC9046292

[50] C. Moore , C. McLister , C. O'Neill , M. Donnelly , and G. McKenna , “Pre‐Radiotherapy Dental Extractions in Patients With Head and Neck Cancer: A Delphi Study,” Journal of Dentistry 97 (2020): 103350, 10.1016/j.jdent.2020.103350. 32371021

[51] C. Hentz , A. Z. Diaz , R. W. Borrowdale , B. Emami , M. Kase , and M. Choi , “Establishing a Targeted Plan for Prophylactic Dental Extractions in Patients With Laryngeal Cancer Receiving Adjuvant Radiotherapy,” Oral Surgery, Oral Medicine, Oral Pathology and Oral Radiology 122, no. 1 (2016): 43–49, 10.1016/j.oooo.2016.01.021. 27068679

[52] S. Hosseinian , M. Hemmati , C. Dede , et al., “Cluster‐Based Toxicity Estimation of Osteoradionecrosis via Unsupervised Machine Learning: Moving Beyond Single Dose‐Parameter Normal Tissue Complication Probability by Using Whole Dose‐Volume Histograms for Cohort Risk Stratification,” International Journal of Radiation Oncology*Biology*Physics 119, no. 5 (2024): 1569–1578, 10.1016/j.ijrobp.2024.02.021. PMC11262961 38462018

[53] L. V. van Dijk , A. A. Abusaif , J. Rigert , et al., “Normal Tissue Complication Probability (NTCP) Prediction Model for Osteoradionecrosis of the Mandible in Patients With Head and Neck Cancer After Radiation Therapy: Large‐Scale Observational Cohort,” International Journal of Radiation Oncology*Biology*Physics 111, no. 2 (2021): 549–558, 10.1016/j.ijrobp.2021.04.042. PMC8906058 33965514

[54] S. Aarup‐Kristensen , C. R. Hansen , L. Forner , C. Brink , J. G. Eriksen , and J. Johansen , “Osteoradionecrosis of the Mandible After Radiotherapy for Head and Neck Cancer: Risk Factors and Dose‐Volume Correlations,” Acta Oncologica 58, no. 10 (2019): 1373–1377, 10.1080/0284186X.2019.1643037. 31364903

[55] A. Singh , S. Kitpanit , B. Neal , et al., “Osteoradionecrosis of the Jaw Following Proton Radiation Therapy for Patients With Head and Neck Cancer,” JAMA Otolaryngology–Head & Neck Surgery 149, no. 2 (2023): 151–159, 10.1001/jamaoto.2022.4165. 36547968 PMC9912132

[56] L. A. de Almeida‐Silva , J. S. Lupp , L. A. Sobral‐Silva , et al., “The Incidence of Osteoradionecrosis of the Jaws in Oral Cavity Cancer Patients Treated With Intensity‐Modulated Radiotherapy: A Systematic Review and Meta‐Analysis,” Oral Surgery, Oral Medicine, Oral Pathology and Oral Radiology 138, no. 1 (2024): 66–78, 10.1016/j.oooo.2024.04.008. 38772792

