# Supplementary material for: Timing for Radiotherapy Initiation After Dental Extraction and Risk of Osteoradionecrosis in Head and Neck Cancer Patients: A Systematic Review and Meta‐Analysis
Source: World J Otorhinolaryngol Head Neck Surg. 2026 Mar 15:10.1002/wjo2.70097. Online ahead of print. doi: 10.1002/wjo2.70097 (PMC13399180; doi:10.1002/wjo2.70097)

Supplementary Materials for search strategies

**Search strategy for PubMed**

| **("Head and Neck Neoplasms"[Mesh] OR "head and neck cancer" [tiab] OR ((head[tiab] OR neck[tiab]) AND (cancer[tiab] OR tumor[tiab] OR tumour[tiab] OR carcinoma*[tiab])))** |
| --- |
| **AND** |
| **(((((((((("tooth extraction") OR "teeth extraction") OR "dental extraction") OR "extraction of teeth") OR "exodontia") OR "exodontics") OR "molar extraction") OR "odontoectomy") OR "tooth removal") OR "tooth resection")** |
| **AND** |
| ("osteoradionecrosis"[MeSH Terms] OR osteoradionecrosis**[tiab]**) |
| **AND** |
| **((("Radiotherapy"[Mesh] OR "radiotherapy" [Subheading] OR "Radiotherapy, Computer-Assisted"[Mesh] OR "Radiotherapy, Image-Guided"[Mesh] OR "Lymphatic Irradiation"[Mesh] OR "Heavy Ion Radiotherapy"[Mesh] OR "Radiosurgery"[Mesh]) OR ("radiation therapy"[tiab]) OR radiation OR radio*))** |

**Search strategy for Ovid-Embase**

| **No.** | **Query** | **Results** |
| --- | --- | --- |
| **#1** | **('osteoradionecrosis'/exp OR 'osteoradionecrosis') AND [embase]/lim** | **2,552** |
| **#2** | **('head' OR 'head'/exp OR head) AND ('neck' OR 'neck'/exp OR neck) AND ('neoplasms' OR 'neoplasms'/exp OR neoplasms) AND [embase]/lim** | **204,605** |
| **#3** | **('head and neck tumor'/exp OR 'head and neck tumor') AND [embase]/lim** | **461,161** |
| **#4** | **('tooth extraction'/exp OR 'tooth extraction') AND [embase]/lim** | **16,197** |
| **#5** | **('radiotherapy'/exp OR radiotherapy) AND [embase]/lim** | **800,699** |
| **#6** | **('radiation'/exp OR 'radiation') AND [embase]/lim** | **1,268,863** |
| **#7** | **#2 or #3** | **506,394** |
| **#8** | **#5 or #6** | **1,627,970** |
| **#9** | **#1 and #4 and #7 and #8** | **198** |

Supplementary Table 1: Characteristics of included studies for meta-analysis

| **Author, year** | **Origin; Design** | **Cancer type and site (number of patients, if avalable)** | **Radiotherapy modality** | **Other therapy** | **Dental extraction time (exposure *vs* control)** | **Dental extraction site、number** |
| --- | --- | --- | --- | --- | --- | --- |
| Starcke, 1977^23^ | Texas, USA; Retrospective cohort study | Cancer type: NA;  Primary site:  Tonsillar pillar (9)  Base of tongue (8)  Larynx (1)  Oral tongue (1)  Retromolar trigone (1)  Pharynx (6)  Nasopharynx (6)  Soft palate (3  Floor of mouth (3)  Mandible (3  Buccal mucosa (1)  Maxillary sinus (1)  Unknown primary (1) | Co^60^ | NA | **<=7 days**  0 ORN case/7 in total  *vs*  **>7 days**  1 ORN case /55 in total | Extraction site: NA;  Tooth number:  <=7 days  10.3±9.19 (mean±sd) teeth  >7 days  15.2±7.98 teeth |
| Wang, 2017^24^ | Taiwan, China; Retrospective cohort study | Cancer type: NA;  Primary site: 24452 patients with buccal, oral cavity, oropharynx, hypopharynx | NA | Surgery  Mandibulectomy  Chemotherapy " | **<=14 days**  815 ORN cases/8756 in total  *vs*  **>14 days**  1083 ORN cases /15796 in total | Extraction site: NA;  Tooth number: extraction in total=10720 |
| Huang, 2020^25^ | Taiwan, China; Retrospective cohort study | Cancer type: NA;  Primary site: Oral cancer (the International Classification of Diseases, Ninth Revision, Clinical Modification (ICD-9-CM) 140–145), | 2000 patients in low dosages (<60 Gy) group, 5107 patients in the high dosages (≥60 Gy) group | Chemotherapy; steroids; aspirin | **<=14 days**  42 ORN cases/1121 in total  *vs*  **>14 days**  58 ORN cases /1752 in total | Extraction site: NA;  Tooth number: NA |
| Liao, 2020^26^ | Taiwan, China; Retrospective cohort study | Cancer type: NA;  Primary site: NA | three-dimensional conformal RT (3D-CRT) before the year of 2009; intensity-modulated  RT (IMRT) and volumetric modulated arc therapy (VMAT) after the year of 2009 | Tumor excision surgery; Chemotherapy; Mandibulectomy; Maxillectomy | **<=7 days**  15 ORN cases/1759 in total  *vs*  **>7days**  37 ORN cases /3303 in total | Extraction site: NA;  Tooth number:  ORN group: 4.3±3.25  Non-ORN4.16±3.39 |
| Chang, 2022^27^ | Taiwan, China; Retrospective cohort study | Cancer type: NA;  Primary site: Oral cancer (ICD-9-CM 140–145) | total RT dose:67.1+-4.6; Adjuvant RT/CRT-276 Neoadjuvant RT/CRT-9 Radical RT/CRT-594 |  | **<=14 days**  20 ORN cases/398 in total  *vs*  **>14 days**  104 ORN cases /1508 in total | Extraction site: NA;  Tooth number:  NA |
| Kovarik, 2022^28^ | Newcastle, UK; Retrospective cohort study | Cancer type: NA;  Primary site: NA | 3D-CRT or IMRT, 55Gy in 20 fractions, 60-65Gy in 30 fractions | No; Cisplatin; or Cetuximab | **<=14 days**  10 ORN cases/79 in total  *vs*  **>14 days**  41 ORN cases /426 in total | Extraction site: NA;  Tooth number:  Mean=6.02 95%CI:(4.72-7.32) |
| Lee, 2023^19^ | Toronto, Canada; Retrospective cohort study | Cancer type: NA;  Primary site:  Larynx (89)  Lip and oral cavity (169)  Oropharynx (298)  Other (323) | Total RT dose:67.1+-4.6; Adjuvant RT/CRT-276 Neoadjuvant RT/CRT-9 Radical RT/CRT-594 | NA | **<=7 days**  19 ORN cases/299 in total  *vs*  **>7days**  13 ORN cases /580 in total  OR  **<=14 days**  30 ORN cases/685 in total  *vs*  **>14 days**  2 ORN cases /194 in total | Extraction site: NA;  Tooth number:  NA |
| Shih, 2024^29^ | Taiwan, China; Retrospective cohort study | Cancer type: NA;  Primary site:  Lip (160)  Tongue (1579)  Gum (856)  Mouth floor (183)  Other parts of oral cavity (2060)  Oropharyngeal (1823)  Nasopharyngeal (3052)  Hypopharyngeal (1281)  Laryngeal (429)  Others part of HNC (266) | IMRT | chemotherapy, target therapy, and surgery | **<=14 days**  108 ORN cases/3572 in total  *vs*  **>14 days**  497 ORN cases /8117 in total | Extraction site: NA;  Tooth number:  NA |

NA: data not available

Supplementary Table 2. Newcastle-Ottawa Scale (NOS) for risk of bias evaluation

| **Study** | **Selection** | | | | **Comparability** | **Outcome** | | | **Summary score** |
| --- | --- | --- | --- | --- | --- | --- | --- | --- | --- |
|  | Representativeness of the exposed cohort | Selection of the non-exposed cohort | Ascertainment of exposure | Demonstration that outcome of interest was not present at start of study | Comparability of cohorts on the basis of the design or analysis | Assessment of outcome | Was follow-up long enough for outcomes to occur | Adequacy of follow up of cohorts |  |
| **Starcke, 1977**^23^ | 1 | 1 | 1 | 1 | 1 | 0 | 1 | 0 | **6** |
| **Wang, 2017**^24^ | 1 | 1 | 1 | 1 | 1 | 1 | 1 | 1 | **8** |
| **Liao, 2020**^25^ | 1 | 1 | 1 | 1 | 1 | 1 | 1 | 1 | **8** |
| **Huang, 2020**^26^ | 1 | 1 | 1 | 1 | 1 | 1 | 1 | 1 | **8** |
| **Chang, 2022**^27^ | 1 | 1 | 1 | 1 | 1 | 1 | 1 | 1 | **8** |
| **Kovarik, 2022**^28^ | 1 | 1 | 1 | 1 | 1 | 1 | 1 | 0 | **7** |
| **Lee, 2023**^19^ | 1 | 1 | 1 | 1 | 2 | 1 | 1 | 1 | **9** |
| **Shih, 2024**^29^ | 1 | 1 | 1 | 1 | 1 | 1 | 1 | 1 | **8** |

Supplementary Figure 1. Funnel plot to assess potential bias from publication.


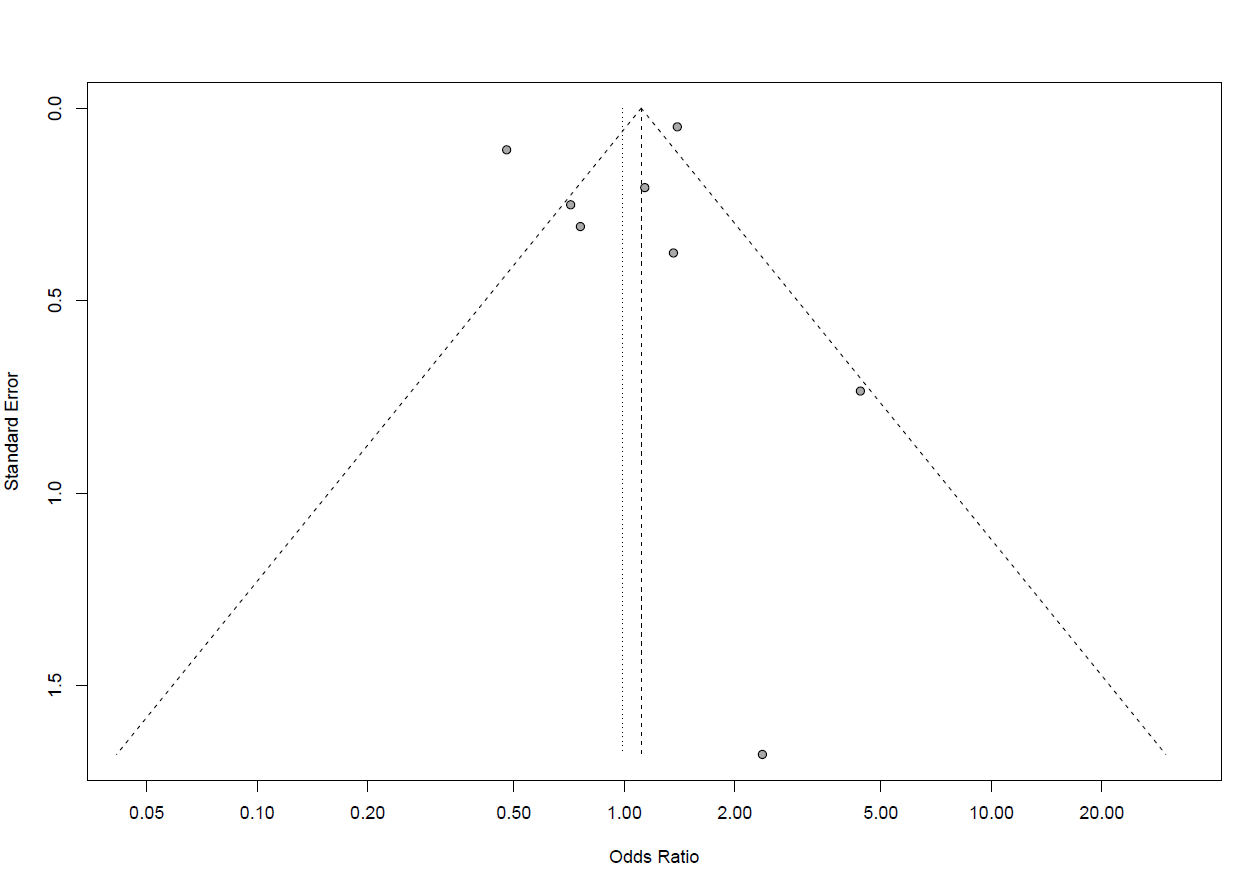


Supplementary Figure 2. Forest plot for sensitivity analysis (leave-one-out) for the association between timing for RT initiation (<=14days vs >14 days) and risk of osteoradionecrosis.


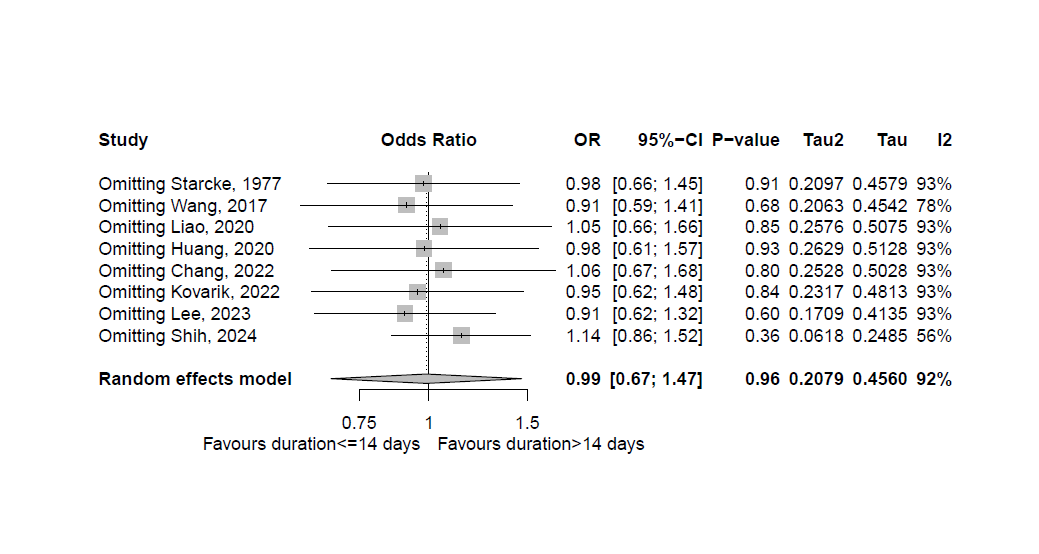

Supplement: Supplementary file 1 — Supplementary Figure 1: Funnel plot to assess potential bias from publication. Supplementary Figure 2: Forest plot for sensitivity analysis (leave‐one‐out) for the association between timing for RT initiation (≤ 14days vs > 14 days) and risk of osteoradionecrosis. Supplementary Table 1: Characteristics of included studies for meta‐analysis. Supplementary Table 2: Newcastle‐Ottawa Scale (NOS) for risk of bias evaluation. [file WJO2-9999-0-s001.docx]
